# Global Value Trees

**DOI:** 10.1371/journal.pone.0126699

**Published:** 2015-05-15

**Authors:** Zhen Zhu, Michelangelo Puliga, Federica Cerina, Alessandro Chessa, Massimo Riccaboni

**Affiliations:** 1 IMT Institute for Advanced Studies Lucca, Lucca, Italy; 2 Linkalab, Complex Systems Computational Laboratory, Cagliari, Italy; 3 Department of Physics, Università degli Studi di Cagliari, Cagliari, Italy; 4 Department of Managerial Economics, Strategy and Innovation, Katholieke Universiteit Leuven, Leuven, Belgium; University of Maribor, SLOVENIA

## Abstract

The fragmentation of production across countries has become an important feature of the globalization in recent decades and is often conceptualized by the term “global value chains” (GVCs). When empirically investigating the GVCs, previous studies are mainly interested in knowing how global the GVCs are rather than how the GVCs look like. From a complex networks perspective, we use the World Input-Output Database (WIOD) to study the evolution of the global production system. We find that the industry-level GVCs are indeed not chain-like but are better characterized by the tree topology. Hence, we compute the global value trees (GVTs) for all the industries available in the WIOD. Moreover, we compute an industry importance measure based on the GVTs and compare it with other network centrality measures. Finally, we discuss some future applications of the GVTs.

## Introduction

The history of globalization has been marked by two great unbundlings, the first being the spatial separation of production and consumption (i.e., international trade in final products), and more recently, the second being the spatial fragmentation within production (i.e., international trade in tasks and supply chains) [1, 2]. The second great unbundling is often conceptualized by the term, global value chains, or GVCs (Other similar concepts used in the literature include global supply chains [3], supply-chain trade [2], international fragmentation [4], outsourcing [5], offshoring [6], and vertical specialization [7].), since it captures the fact that the value-added of a final product can be distributed globally. In other words, a product (and its components) may have crossed multiple country borders before it arrives in a final consumer’s hands. For instance, before it hits the US market, an Apple’s iPod needs to be assembled in China, which in turn sources microchips and software from Japan, South Korea, and the US itself [8].

Quite a few theoretical models have been developed to understand the GVCs’ structure, mechanism, welfare impacts, and policy implications [3, 6, 9]. Thanks to the recently constructed global multi-regional input-output (MRIO) tables, empirical studies can be conducted at the industry level and hence identify a more general pattern of the GVCs than do the case studies on the specific products such as iPod. In particular, the global value-added content of exports for a given industry or country can be measured [2, 4, 5, 7, 10–12].

Although previous studies can tell us how global the GVCs are, very little is known about how the GVCs look like (A fairly comprehensive survey of the GVCs literature is conducted by Amador and Cabral [13]. There are a number of studies exploring some structural properties of the GVCs such as the length of a GVC and the industry upstreamness with respect to final consumption [14, 15]. However, they only provide some rough estimates of the structural properties rather than any topological details of the GVCs.). To fill the gap in the literature, our paper is the first attempt to investigate the topological properties of the industry-level GVCs. From a complex networks perspective, we map the World Input-Output Database (WIOD) into the global value networks (GVNs), where the nodes are the individual industries in different countries and the edges are the value-added contribution relationships.

Based on the GVNs, this paper makes some significant contributions to the literature of the GVCs. First, unlike the previous literature which provides only some rough estimates of the structural properties of the GVCs, we are able to produce a detailed topological view of the industry-level GVCs. We compute the global value trees (GVTs) for all the industries available in the WIOD by a breadth-first search algorithm with the edge direction and a threshold of edge weight. We explore some basic properties of the GVTs. In particular, we estimate the allometric scaling exponents and verify that the GVTs are topologically between a star and a chain. Second, we develop an industry importance measure based on the GVTs and compare it with other network centrality measures of the industries. We find that the tree-based measure performs the best in terms of the correlation with the industry total value-added. Therefore, the GVTs still retain the essential information of the GVNs and can be viewed as a reasonable simplification of the latter. Third, with the rich topological information, the GVTs enable a broad range of empirical studies of the global fragmentation of production such as to examine the evolution of the GVTs for a certain industry and to compare the GVTs of the same industry in different countries.

The rest of the paper is structured as follows. Section 2 maps the WIOD database into the GVNs and develops an algorithm to compute the GVTs. Section 3 explores some basic properties of the GVTs. In particular, we quantify the allometric scaling pattern of the GVTs and propose an industry importance measure based on the GVTs and compare it with other network centrality measures. Finally, Section 4 discusses some future applications of the GVTs and concludes the paper.

## Methods

The complex networks approach has been widely used in economics and finance in recent years [16–24]. Designed to keep track of the inter-industrial relationships, the input-output system is an ideal test bed for network science. In particular, the global MRIO system can be viewed as an interdependent complex network [25], where the nodes are the individual industries in different countries and the edges are the input-output relationships between industries [24].

This paper takes one step further and uses the WIOD database to construct the global value networks (GVNs), where the nodes are the individual industries in different countries and the edges are the value-added contribution relationships (The call for a network analysis of the GVCs has existed for years [26–29].). Moreover, based on the GVNs, the global value trees (GVTs) can be computed in a straightforward manner.

### Data Description

We use the World Input-Output Database (WIOD) [30] to compute the GVNs and the GVTs. At the time of writing, the WIOD input-output tables cover 35 industries for each of the 40 economies (27 EU countries and 13 major economies in other regions) plus the rest of the world (RoW) and the years from 1995 to 2011 (The 40 economies are representative of the world economy in a sense that they produce around 84.1% of the world GDP in 2011. S1 and S2 Tables have the lists of countries and industries covered in the WIOD.). For each year, there is a harmonized global level input-output table recording the input-output relationships between any pair of industries in any pair of economies. The numbers in the WIOD are in current basic (producers’) prices and are expressed in millions of US dollars (The basic prices are also called the producers’ prices, which represent the amount receivable by the producers. An alternative is the purchases’ prices, which represent the amount paid by the purchases and often include trade and transport margins. The former is preferred by the WIOD because it better reflects the cost structures underlying the industries [30].). Table 1 shows an example of a global MRIO table with two economies and two industries. The 4 × 4 inter-industry table is called the transactions matrix and is often denoted by **Z**. The rows of **Z** record the distributions of the industry outputs throughout the two economies while the columns of **Z** record the composition of inputs required by each industry. Notice that in this example all the industries buy inputs from themselves, which is often observed in real data. Besides intermediate industry use, the remaining outputs are absorbed by the additional columns of final demand, which includes household consumption, government expenditure, and so forth (In Table 1 we only show the aggregated final demand for the two economies.). Similarly, production necessitates not only inter-industry transactions but also labor, management, depreciation of capital, and taxes, which are summarized as the additional row of value-added. The final demand matrix is often denoted by **F** and the value-added vector is often denoted by **v**. Finally, the last row and the last column record the total industry outputs and its vector is denoted by **x**.

**Table 1:** A hypothetical two-economy-two-industry MRIO table. The 4 × 4 inter-industry transactions matrix records outputs selling in its rows and inputs buying in its columns. The additional columns are the final demand and the additional row is the value added. Finally, the last column and the last row record the total industry outputs.

		Buyer Industry						
		Economy 1		Economy 2		Final Demand		
Seller Industry		Industry 1	Industry 2	Industry 1	Industry 2	Economy 1	Economy 2	Total Output
Economy 1	Industry 1	25	10	20	10	45	10	120
	Industry 2	10	5	10	20	50	5	100
Economy 2	Industry 1	30	15	600	500	5	8650	9800
	Industry 2	35	30	1000	1000	25	7910	10000
Value Added		20	40	8170	8470			
Total Output		120	100	9800	10000			

### Construct the Global Value Networks

If we use **i** to denote a summation vector of conformable size, i.e., a vector of all 1’s with the length conformable to the multiplying matrix, and let **Fi** = **f**, we then have **Zi**+**f** = **x**. Furthermore, if dividing each column of **Z** by its corresponding total output in **x**, we get the so-called technical coefficients matrix **A** (The ratios are called technical coefficients because they represent the technologies employed by the industries to transform inputs into outputs.). Replacing **Zi** with **Ax**, we rewrite the above equation as **Ax**+**f** = **x**. It can be rearranged as (**I**−**A**)**x** = **f**. Then we can solve **x** as follows:

$$\begin{array}{c} x={\left(I-A\right)}^{-1}f \end{array}$$ (1)

where matrix (**I**−**A**)^−1^ is often denoted by **L** and is called the Leontief inverse [31, 32].

If dividing each element of **v** by its corresponding total output in **x**, we get the value-added share vector and denote it by **u**. Moreover, if we use $\hat{u}$ to denote a diagonal matrix with **u** on its diagonal, then the value-added contribution matrix can be computed as follows:

$$\begin{array}{c} G=\hat{u}L \end{array}$$ (2)

where **G** is the value-added contribution matrix and its element 0 ≤ *G*_*ij*_ ≤ 1 is industry *i*’s share of the value-added contribution in industry *j*’s final demand, *f*_*j*_.

Finally, the GVNs can be constructed by using **G** as the adjacency matrix. Notice that the GVNs are both directed and weighted (We don’t consider the self-loops so that we replace the diagonal of **G** with zeros. Meanwhile, we don’t consider the rest of the world (RoW) and focus our attention on the 40 countries available in the WIOD.).

### Compute the Global Value Trees

Based on the GVNs, the GVTs can be obtained by a modified breadth-first search (BFS) algorithm. Rather than implementing a BFS on the whole network based on a random root, each time we initiate a BFS from a different industry and eventually we build the GVTs for all the industries available in the WIOD. To ensure that the GVTs are topologically different from each other and that each GVT contains only the most essential value-added contribution relationships for the root industry, our BFS algorithm is governed by both the edge direction and a threshold of edge weight. The description of our algorithm is as follows. For each industry available in the WIOD, we first choose the industry as the root of the GVT and initiate the BFS. At each step, the new nodes are added based on their value-added contributions to the existing nodes. As a result, each GVT captures the value-added flows from the leaf industries to the root industry. Second, since the GVNs are almost completely connected (This is a general feature of the input-output networks due to the aggregated industry classification [24].), we search the GVTs based on a threshold of the edge weight, which we denote by *α*, in order to keep only the most essential value-added contribution relationships for the root industry.

**Fig 1:**
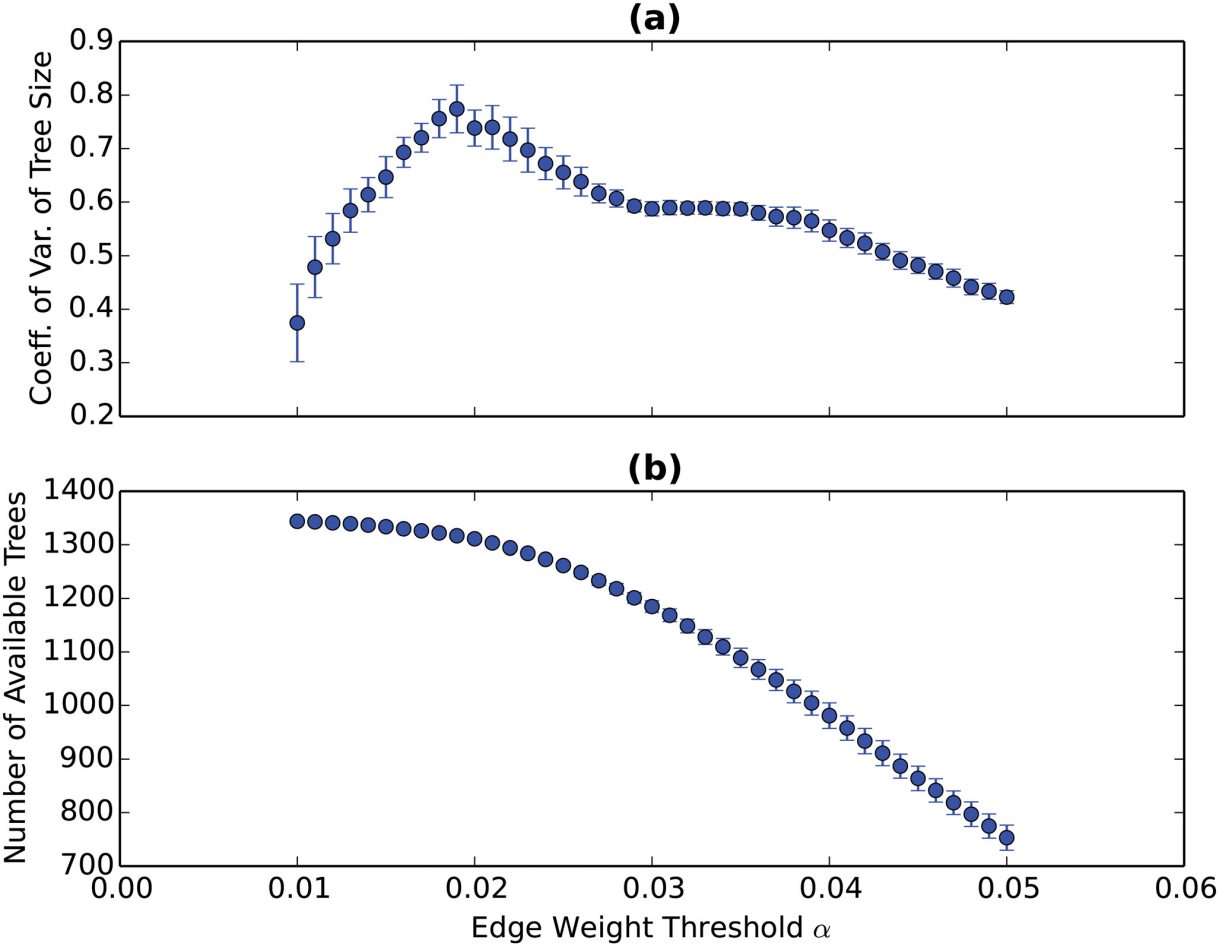
The impact of *α* on the GVTs obtained. Panel (a) shows the relationship between the tree size variation and the value of *α* while panel (b) shows the relationship between the number of available GVTs and the value of *α*. The 95% confidence intervals are based on the time variation during 1995-2011. The maximum variation of tree size is obtained when *α* = 0.019. The number of available GVTs according to this value of *α* is still very large (around 1300) given that the total number of industries in the WIOD is 1400.

To determine a benchmark value of *α*, we take into account two empirical relationships between *α* and the GVTs obtained. First, as the value of *α* increases, the number of available (nonempty) GVTs will decrease since it becomes more difficult for a value-added contribution to pass the threshold. Second, we examine the relationship between the value of *α* and the size (as measured by the total number of nodes in a GVT) variation across the obtained GVTs. A larger variation of tree size is more desirable because the topological differences between the GVTs can be better revealed if the GVTs obtained are more diverse in terms of tree size. Therefore, the optimal value of *α* can be obtained when the tree size variation is maximized and when the number of available GVTs is reasonably large. The two empirical relationships are plotted in Fig 1. Panel (a) shows the relationship between the tree size variation and the value of *α* and the relationship is not monotonic but has a clear optimum. Panel (b) shows the relationship between the number of available GVTs and the value of *α* and they are indeed inversely related. The maximum variation of tree size is obtained when *α* = 0.019. The number of available GVTs according to this value of *α* is still very large (around 1300) given that the total number of industries in the WIOD is 1400. Hence, the benchmark value of *α* is determined to be 0.019.

**Fig 2:**
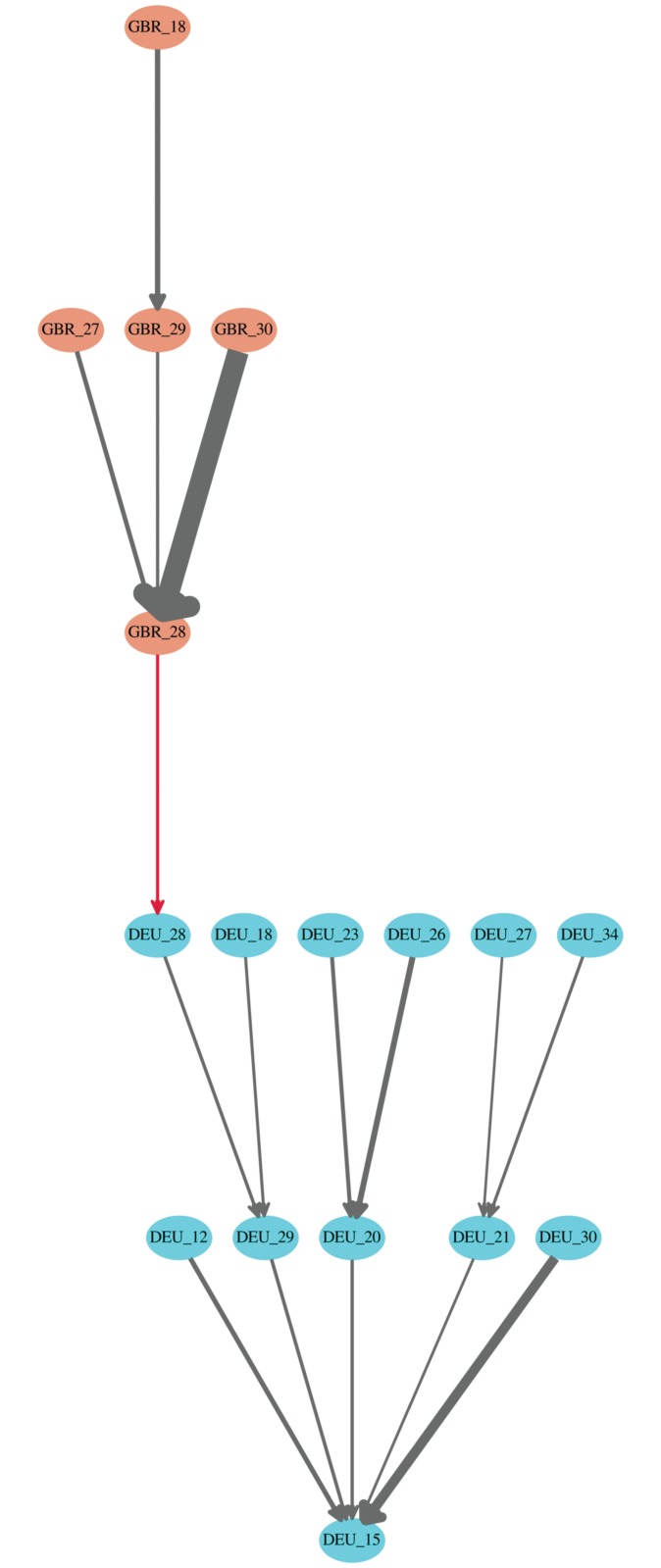
The GVT rooted at Germany’s transport equipment industry (DEU_15) in 2011. The edge weight threshold is set to 0.019. Different colors of the nodes indicate different countries. The red edges indicate cross-country relationships while the gray edges indicate domestic relationships. The edge width is proportional to the edge weight, i.e., the share of the value-added contribution. The codes of countries and industries can be found in S1 and S2 Tables.

As an example of the GVTs based on *α* = 0.019, Fig 2 shows the GVT rooted at Germany’s transport equipment industry (DEU_15) in 2011. Different colors of the nodes indicate different countries. The red edges indicate cross-country relationships while the gray edges indicate domestic relationships. The edge width is proportional to the edge weight, i.e., the share of the value-added contribution. In this example, the first two steps of our BFS algorithm have added some other domestic industries in Germany. At the third step, UK’s financial intermediation industry (GBR_28) is added as a significant value-added contributor to Germany’s financial intermediation industry (DEU_28). Finally, governed by the edge direction and the threshold of edge weight (*α* = 0.019), the search is completed at the fifth step with UK’s construction industry (GBR_18).

## Results

Once we have computed the GVTs, some basic properties of the tree topology can be explored. Subsection 3.1 quantifies the allometric scaling pattern of the GVTs. We estimate the allometric scaling exponents and verify that the GVTs are topologically between a star and a chain. Subsection 3.2 proposes a tree-based industry importance measure and compares it with other network centrality measures. We find that the tree-based measure performs the best in terms of the correlation with the industry total value-added. Therefore, the GVTs still retain the essential information of the GVNs and can be viewed as a reasonable simplification of the latter.

### Allometric Scaling Pattern

The allometric scaling pattern refers to the power law relationship between size and other physical or behavioral variables. Previous studies have documented the ubiquitous existence of the allometric scaling pattern in systems as diverse as river networks, cellular metabolism, population dynamics, and food web [33, 34].

For a directed tree topology, if we denote the total number of nodes in the sub-tree rooted at node *i* by *X*_*i*_ and the sum of all *X*_*i*_’s in the sub-tree rooted at node *i* by *Y*_*i*_, then an allometric scaling relationship is observed between *Y*_*i*_ and *X*_*i*_ and can be described by a power law, i.e., $Y_{i}∼X_{i}^{\eta }$, where *η* is called the allometric scaling exponent.

**Fig 3:**
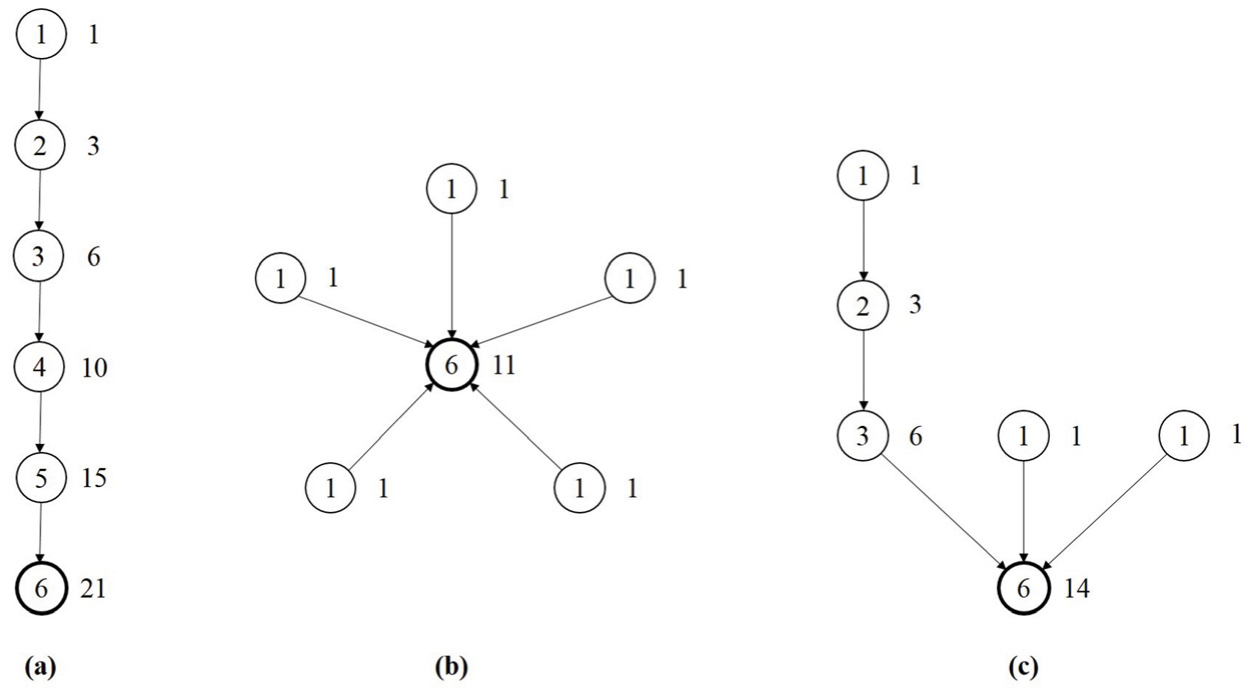
Examples of the allometric scaling relationship. The numbers inside the node circles are *X*_*i*_’s whereas those next to the circles are *Y*_*i*_’s. The node with the thick circle is the root. From left to right, they are a chain (a), a star (b), and a tree (c), respectively.

Fig 3 shows the examples of a chain, a star, and a tree, respectively. The numbers inside the node circles are *X*_*i*_’s whereas those next to the circles are *Y*_*i*_’s. The allometric scaling exponent *η* of a tree is lower-bounded by that of a star (*η* = 1) and upper-bounded by that of a chain (*η* = 2). As a result, *η* can be interpreted as a measure of hierarchicality, as star is the “flattest” topology and chain is the most hierarchical topology given the same number of nodes.

**Fig 4:**
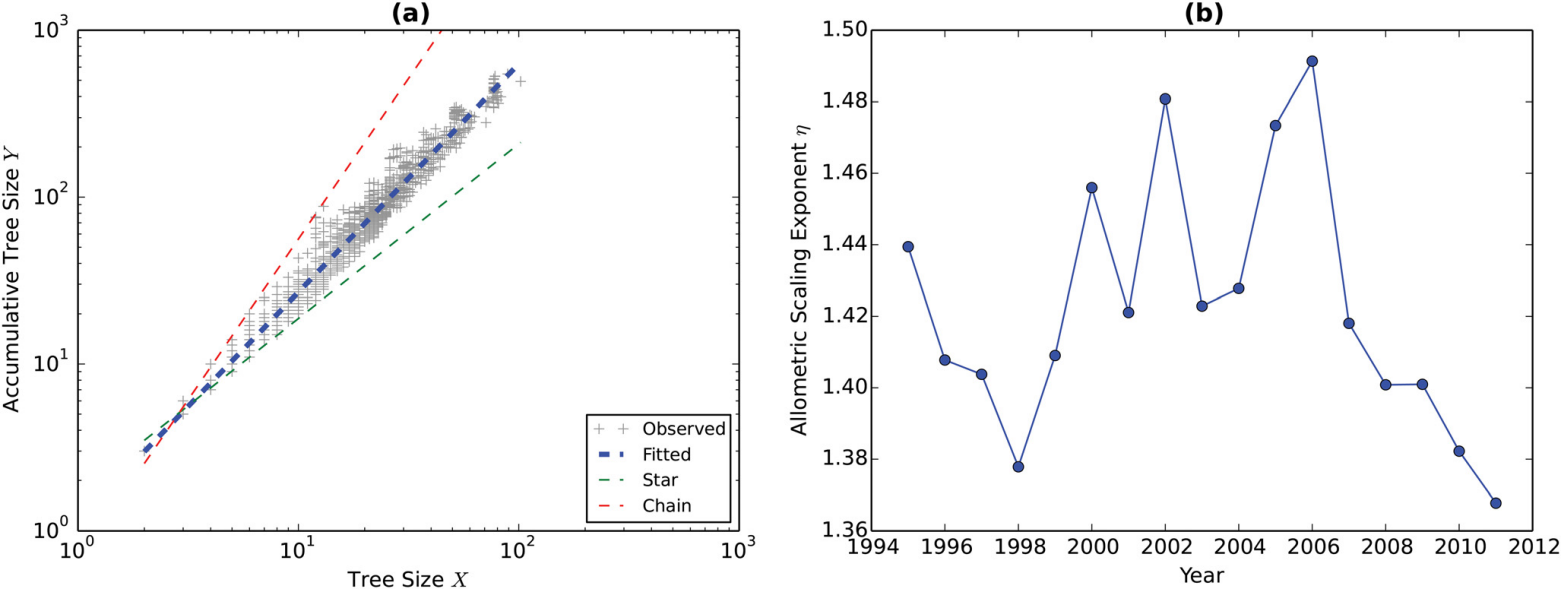
Estimation of the allometric scaling exponent *η*. Panel (a) shows the log-log plot of the root-node *Y*_*i*_-*X*_*i*_ pairs in 2011, where the horizontal axis is the *X*_*i*_ of the root node, i.e., the total number of nodes in a given GVT (the tree size), and the vertical axis is the *Y*_*i*_ of the root node, which we call the accumulative tree size. The gray crosses are the observed data points. The thick blue dashed line is fitted with the observed data and with the slope of *η*. The fitting lines for star and chain based on the same set of *X*_*i*_’s are the green dashed line and the red dashed line respectively. Panel (b) plots the estimated *η*’s over time.

To examine the hierarchicality of the GVTs, we estimate *η*’s based on the root-node *Y*_*i*_-*X*_*i*_ pairs across all the GVTs for each year. Fig 4 has the estimation result of *η*. Panel (a) shows the log-log plot of the root-node *Y*_*i*_-*X*_*i*_ pairs in 2011, where the horizontal axis is the *X*_*i*_ of the root node, i.e., the total number of nodes in a given GVT (the tree size), and the vertical axis is the *Y*_*i*_ of the root node, which we call the accumulative tree size. The gray crosses are the observed data points. The thick blue dashed line is fitted with the observed data and with the slope of *η*. The fitting lines for star and chain based on the same set of *X*_*i*_’s are the green dashed line and the red dashed line respectively. It is straightforward to see that in 2011 the GVTs are topologically between a star than to a chain. Panel (b) plots the estimated *η*’s over time. The values of *η* are between 1.36 and 1.5 for all the years (Shi et al. [35] also estimate the allometric scaling exponent to understand the hierarchicality of the global production system. However, they consider the directed tree as a flow network. Furthermore, their paper differs from ours in both data source and research strategy. They use the United Nations COMTRADE database to construct the product-specific trade networks while we use the WIOD database to construct the GVNs with both country and industry dimensions.).

### A Tree-Based Importance Measure

The GVTs are the subgraphs of the GVNs. Unlike the GVNs, the GVTs reveal the local importance of the industries. Previous studies have shown that the subgraph centrality measure can be used to complement the global centrality measures [36]. Hence, we compute a simple industry importance measure based on the GVTs and compare it with other network centrality measures.

First, we denote a tree with the root *r* by *T*(*r*). Furthermore, we denote the total number of nodes in the sub-tree rooted at industry *i* by *X*_*i*_(*r*) and the total number of nodes in the tree *T*(*r*) by *N*(*r*). If industry *i* is present in *k* trees all over the world and we denote the set of roots of the *k* trees by *S*_*i*_, then the importance of industry *i* is defined as follows:

$$\begin{array}{c} TI_{i}=\sum_{r\in S_{i},r\neq i}\frac{X_{i}\left(r\right)}{N(r)}\frac{FD(r)}{WGDP} \end{array}$$ (3)

where *TI*_*i*_ is the tree-based importance measure of industry *i*, *FD*(*r*) is the final demand in the root industry *r* (i.e., total production of the root industry *r* minus its intermediate supply to other industries according to the WIOD input-output table) and *WGDP* is the world GDP (i.e., summing up the final demand of all the industries around the world according to the WIOD input-output table). Notice that when calculating *TI*_*i*_, we don’t consider the role played by industry *i* in its own GVT (i.e., *r* ≠ *i*), although the input-output network has strong self-loops [24].

The economic interpretation of the importance measure is that, more important industries are more closely attached to the root and are able to “pull” a larger portion of the GVTs (measured by $\frac{X_{i}(r)}{N(r)}$) and are associated with more important roots (measured by $\frac{FD(r)}{WGDP}$).

**Fig 5:**
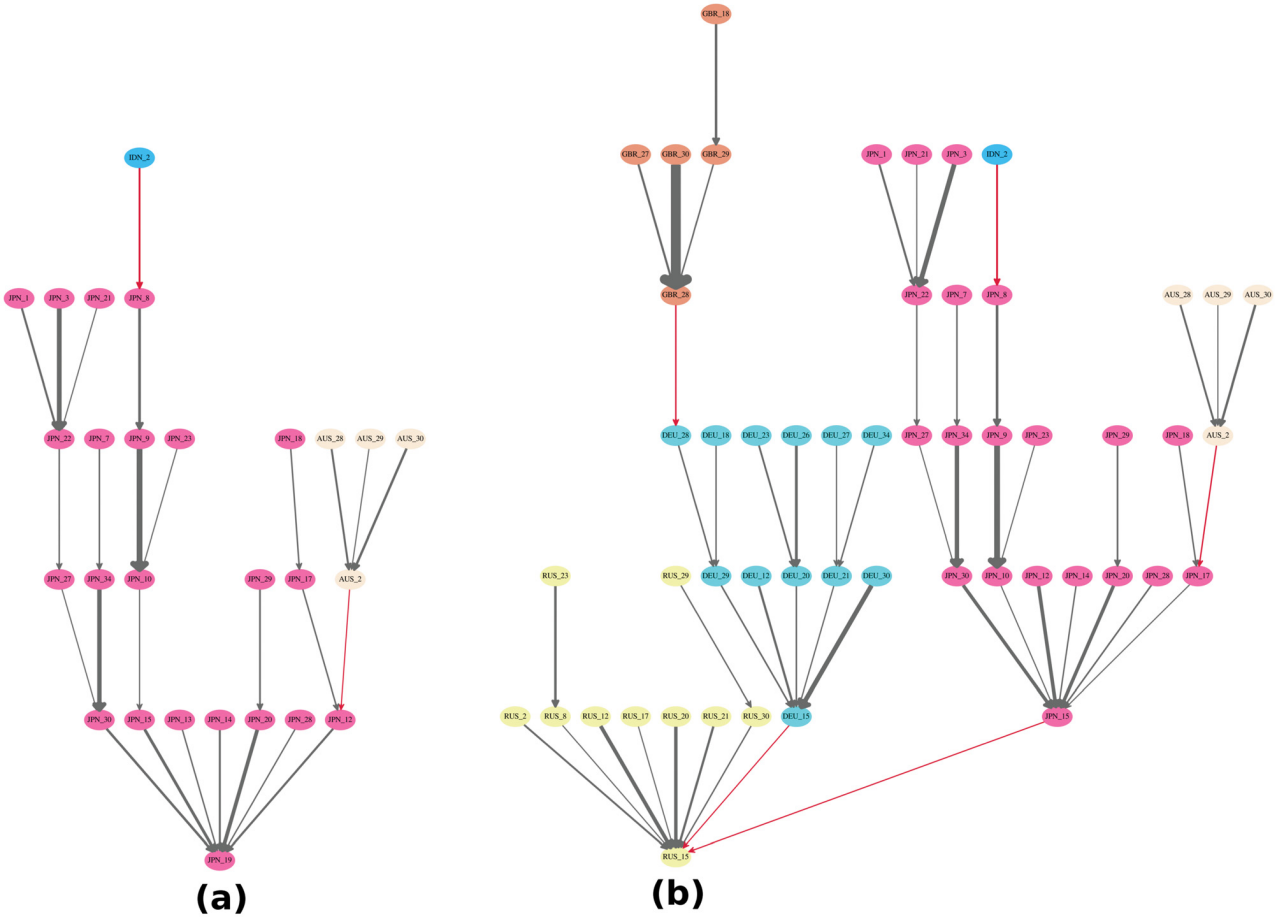
The domestic and foreign GVTs where Japan’s transport equipment industry (JPN_15) has the highest importance score in 2011. Panel (a) shows the domestic GVT where Japan’s transport equipment industry (JPN_15) has the highest importance score while panel (b) shows the foreign GVT where Japan’s transport equipment industry (JPN_15) has the highest importance score. The edge weight threshold is set to 0.019. Different colors of the nodes indicate different countries. The red edges indicate cross-country relationships while the gray edges indicate domestic relationships. The edge width is proportional to the edge weight, i.e., the share of the value-added contribution. High-resolution plots for both panels can be found in S1 and S2 Figs. The codes of countries and industries can be found in S1 and S2 Tables.

Moreover, since each *T*(*r*) where industry *i* is present has a score of importance, i.e., $\frac{X_{i}(r)}{N(r)}\frac{FD(r)}{WGDP}$, we can identify the GVTs where industry *i* has the highest importance score. For instance, Fig 5 shows the GVTs where Japan’s transport equipment industry (JPN_15) has the highest importance score for domestic and foreign roots respectively in 2011.

To examine the tree-based importance measure in a more systematic way, we compare it with other network centrality measures. Table 2 reports the Pearson correlation coefficients among them (in logarithm) for the selected years. For a given year, all the coefficients are based on a common sample among the different measures. It turns out that all the coefficients are positive and almost all of them are significant at 1% level (The only exceptions are between *log*(*BC*) and *log*(*CC*) in 2003 and 2011.). Moreover, S3 Table has the top-20 industries identified by different measures for the selected years while S4 Table reports the country rankings by summing up the measures of the industries in the same country.

**Table 2:** The Pearson correlation coefficient matrix between the tree-based importance measure and other network centrality measures (in logarithm) for the selected years. The size of the sample is in the parentheses next to the corresponding years. *TI* is the tree-based importance measure, *CC* is the closeness centrality, *PR* is the PageRank centrality, *BC* is the betweenness centrality, and *VT* is the industry total value-added. ** means that the coefficient is significant at 1% level. ** means that the coefficient is significant at 1% level.

**1995** (# Obs. 396)						**2003** (# Obs. 356)						**2011** (# Obs. 332)					
	*log*(*TI*)	*log*(*CC*)	*log*(*PR*)	*log*(*BC*)	*log*(*VT*)		*log*(*TI*)	*log*(*CC*)	*log*(*PR*)	*log*(*BC*)	*log*(*VT*)		*log*(*TI*)	*log*(*CC*)	*log*(*PR*)	*log*(*BC*)	*log*(*VT*)
*log*(*TI*)	1	-	-	-	-	*log*(*TI*)	1	-	-	-	-	*log*(*TI*)	1	-	-	-	-
*log*(*CC*)	0.574**	1	-	-	-	*log*(*CC*)	0.543**	1	-	-	-	*log*(*CC*)	0.480**	1	-	-	-
*log*(*PR*)	0.350**	0.373**	1	-	-	*log*(*PR*)	0.414**	0.392**	1	-	-	*log*(*PR*)	0.354**	0.320**	1	-	-
*log*(*BC*)	0.278**	0.171**	0.355**	1	-	*log*(*BC*)	0.229**	0.058	0.283**	1	-	*log*(*BC*)	0.261**	0.075	0.209**	1	-
*log*(*VT*)	0.763**	0.718**	0.658**	0.315**	1	*log*(*VT*)	0.747**	0.733**	0.664**	0.229**	1	*log*(*VT*)	0.758**	0.651**	0.609**	0.262**	1

We find that *TI* performs the best in terms of the correlation with *VT*. Nevertheless, this is not to say that we should abandon other measures and solely use *TI* to understand the importance of a given industry. After all, we only consider the intermediate value-added flows when calculating *CC*, *BC*, and *PR*, whereas we also take into account the final demand in the root industry, i.e., *FD*(*r*), when calculating *TI*, which gives more power to *TI* in explaining *VT*. However, the strong correlation between *log*(*TI*) and *log*(*VT*) at least shows that the GVTs retain the essential information of the GVNs and can be viewed as a reasonable simplification of the latter. That is, *TI* can be considered as a measure of industry’s position advantage. An industry holds an advantageous position by either attaching to big industries (i.e., big $\frac{FD(r)}{WGDP}$) or by affecting big portion of the GVTs (i.e., big $\frac{X_{i}(r)}{N(r)}$). As a result, the better-positioned industries are more competitive in the world production system and hence are able to extract more value-added across the GVTs. Moreover, since the component $\frac{X_{i}(r)}{N(r)}$ of *TI* measures how closely the given industry is attached to the roots (i.e., bigger $\frac{X_{i}(r)}{N(r)}$ implies smaller distance to the roots), it can be considered as a measure of downstreamness. That is, the higher *TI* is the more downstream the industry is in the GVTs. Therefore, the strong correlation between *log*(*TI*) and *log*(*VT*) supports Stan Shih’s theory of “smiling curve”, which states that most value-added potentials are concentrated at the beginning (upstream) and the ending (downstream) parts of the supply chains.

## Discussion

**Fig 6:**
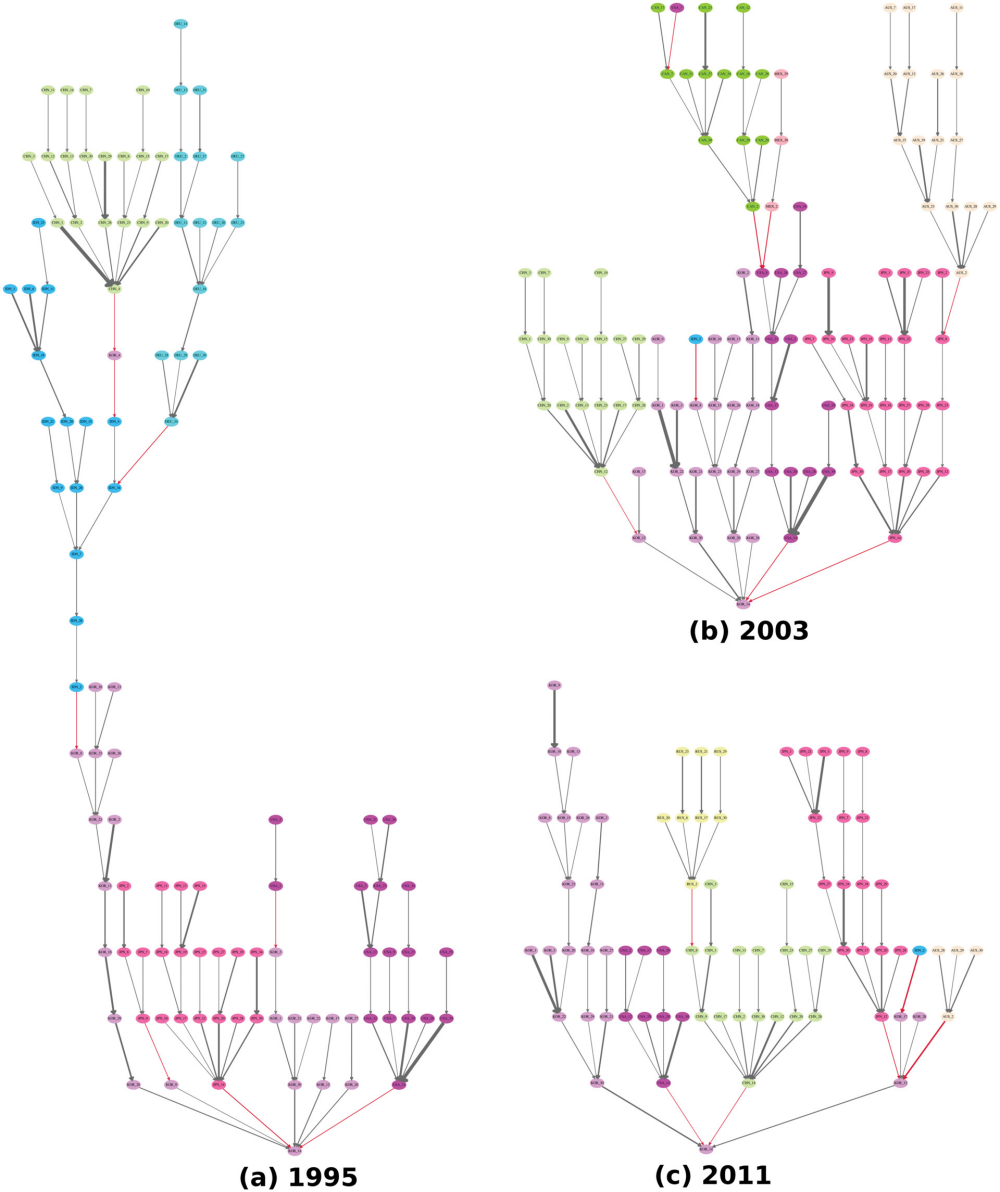
The evolution of the GVTs rooted at South Korea’s electrical equipment industry (KOR_14). Panels (a), (b), and (c) show the GVTs for 1995, 2003, and 2011 respectively. The edge weight threshold is set to 0.019. Different colors of the nodes indicate different countries. The red edges indicate cross-country relationships while the gray edges indicate domestic relationships. The edge width is proportional to the edge weight, i.e., the share of the value-added contribution. China’s cluster is with light green color. High-resolution plots for all the panels can be found in S3, S4 and S5 Figs. The codes of countries and industries can be found in S1 and S2 Tables.

**Fig 7:**
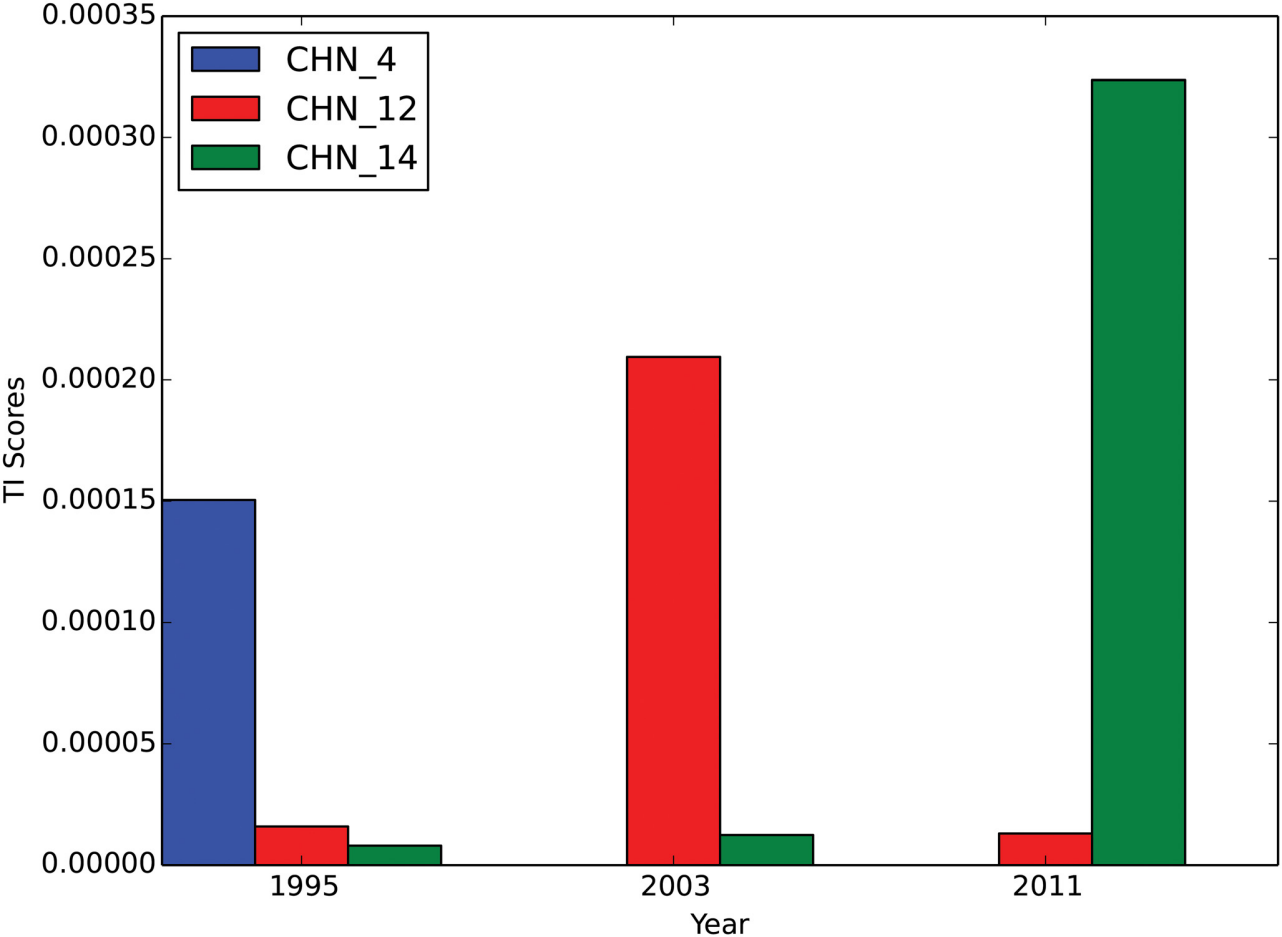
The *TI* scores of China’s indutries in the GVTs rooted at South Korea’s electrical equipment industry (KOR_14). The *TI* scores of three China’s industries in the GVTs rooted at South Korea’s electrical equipment industry are compared over the years. China’s textiles industry (CHN_2; blue bars) has the highest *TI* score among the three in 1995 and disappears in the GVTs in 2003 and 2011. China’s metals industry (CHN_12; red bars) has the highest *TI* score among the three in 2003 but decreases again in 2011. China’s electrical equipment industry (CHN_14; green bars) has increased its *TI* score over time and has the highest *TI* score among the three in 2011.

Once we have the GVTs computed for all the industries available in the WIOD, many interesting questions can be proposed and answered. For instance, does a tree with a fixed root grow over time? This question can be answered by fixing the root industry and examining the GVTs over time. As an example, Fig 6 shows the evolution of the GVTs rooted at South Korea’s electrical equipment industry (KOR_14) over time. There are obviously many interesting structural changes in this example. The most interesting one we find is the dynamics of China’s cluster (with light green color) in the GVTs. In 1995, China’s cluster is relatively far from the root industry and it is connected through China’s textiles industry (CHN_4). In 2003, China’s cluster moves much closer to the root industry and it is connected through China’s metals industry (CHN_12), which is only two steps (degrees) away from the root. Finally, in 2011, China’s cluster is attached directly to the root industry and it is connected through China’s electrical equipment industry (CHN_14). Another way to examine the evolution of China’s cluster is to calculate the *TI* scores for each China’s industry in the GVTs. Fig 7 shows the *TI* scores of three China’s industries (textiles, metals, and electrical equipment) over time in the GVTs in Fig 6. It is straightforward to see that the importance of China’s textiles industry (CHN_4) has been decreasing while the importance of China’s electrical equipment industry (CHN_14) has been increasing according to their *TI* scores. Therefore, our *TI* measure well captures the dynamics of the relative importance of nodes in the GVTs. In this example, the importance of China’s cluster with respect to South Korea’s electrical equipment industry (KOR_14) has been increasing over time. More importantly, some industrial upgrading must have happened in China so that the critical connector switches from China’s textiles industry (CHN_4) to its electrical equipment industry (CHN_14).

**Fig 8:**
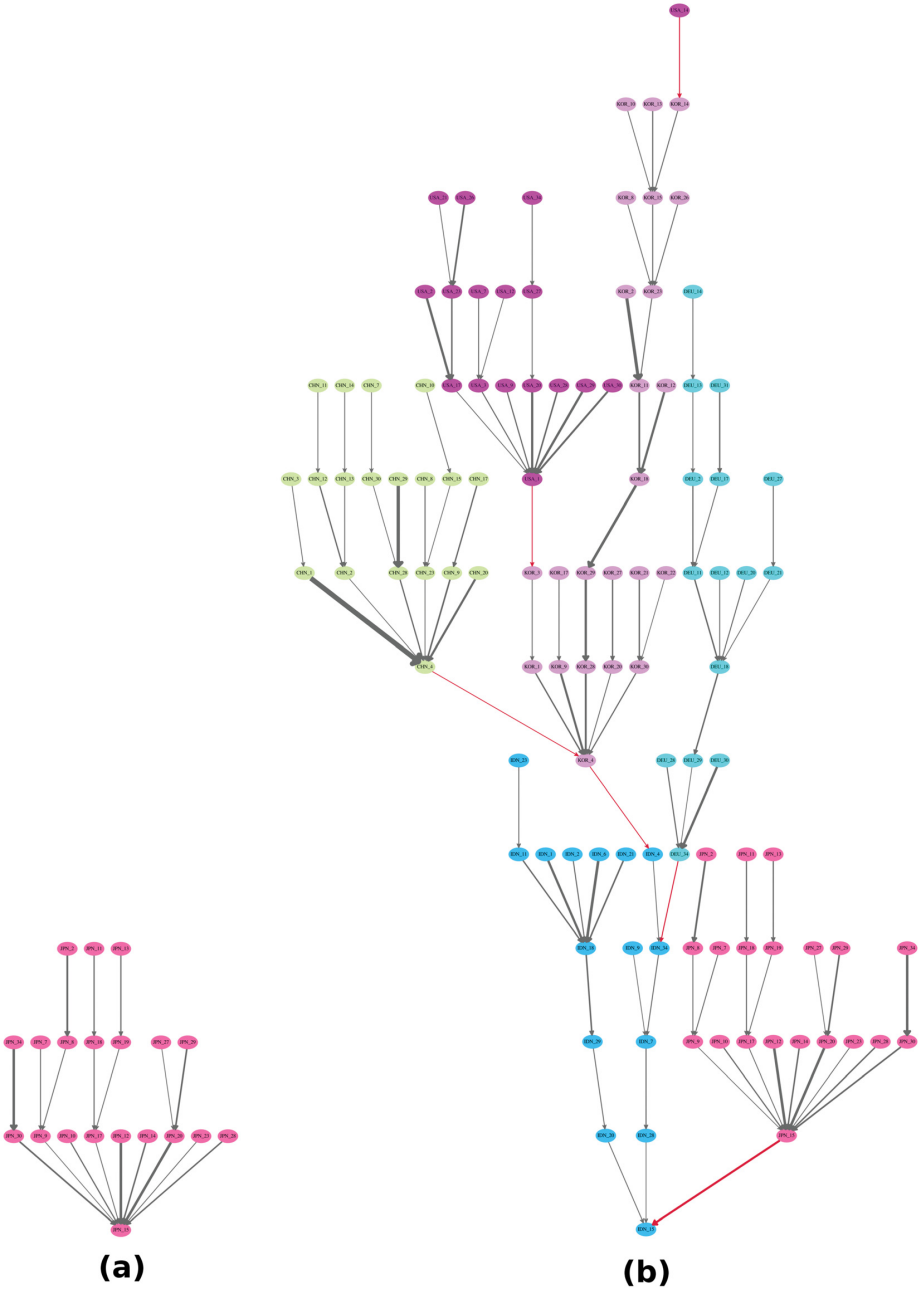
The comparison of the transport equipment industry between Indonesia (IDN_15) and Japan (JPN_15) in 1995. In panel (a) and panel (b), the GVTs are rooted at the tranport equipment industry in Indonesia (IDN_15) and Japan (JPN_15) respectively in 1995. The edge weight threshold is set to 0.019. Different colors of the nodes indicate different countries. The red edges indicate cross-country relationships while the gray edges indicate domestic relationships. The edge width is proportional to the edge weight, i.e., the share of the value-added contribution. High-resolution plots for both panels can be found in S6 and S7 Figs. The codes of countries and industries can be found in S1 and S2 Tables.

We can also examine the different structures of the GVTs for the same industry and the same year but for different countries. Fig 8 compares the transport equipment industry between Indonesia (IDN_15) and Japan (JPN_15) in 1995. The immediate conclusion from this comparison is that the transport equipment industry has a more international GVT in Indonesia than in Japan. More interestingly, Japan’s industries actually play important roles in Indonesia’s GVT, i.e., Japan’s cluster is attached directly to the root in Indonesia and it is connected through Japan’s transport equipment industry (This observation coincides with the increased foreign direct investment from Japan to Indonesia’s car industry in 1995.). In this simple comparison, Japan’s transport equipment industry (JPN_15) is clearly more competitive than Indonesia’s transport equipment industry (IDN_15), according to the above *TI* measure.

In summary, previous studies of the GVCs are mainly interested in knowing how global the GVCs are rather than how the GVCs look like. To fill the gap in the literature, our paper is the first attempt to investigate the topological properties of the industry-level GVCs. From a complex networks perspective, we map the World Input-Output Database (WIOD) into the global value networks (GVNs), where the nodes are the individual industries in different countries and the edges are the value-added contribution relationships.

Based on the GVNs, the global value trees (GVTs) can be obtained by a breadth-first search algorithm with the edge direction and a threshold of edge weight. We compute the GVTs for all the industries available in the WIOD and explore some basic properties of the GVTs. In particular, we estimate the allometric scaling exponents and verify that the GVTs are topologically between a star and a chain. We also develop an industry importance measure based on the GVTs and compare it with other network centrality measures of the industries. We find that the tree-based measure performs the best in terms of the correlation with the industry total value-added. Therefore, the GVTs still retain the essential information of the GVNs and can be viewed as a reasonable simplification of the latter. Finally, we discuss some future applications of the GVTs such as to examine the evolution of the GVTs for a certain industry and to compare the GVTs of the same industry in different countries.

## Supporting Information

S1 Fig The GVT rooted at Japan’s sales of motor vehicles industry in 2011. The edge weight threshold is set to 0.019. Different colors of the nodes indicate different countries. The red edges indicate cross-country relationships while the gray edges indicate domestic relationships. The edge width is proportional to the edge weight, i.e., the share of the value-added contribution. The codes of countries and industries can be found in S1 and S2 Tables.
(PDF)

S2 Fig The GVT rooted at Russia’s transport equipment industry in 2011. The edge weight threshold is set to 0.019. Different colors of the nodes indicate different countries. The red edges indicate cross-country relationships while the gray edges indicate domestic relationships. The edge width is proportional to the edge weight, i.e., the share of the value-added contribution. The codes of countries and industries can be found in S1 and S2 Tables.
(PDF)

S3 Fig The GVT rooted at South Korea’s transport equipment industry in 1995. The edge weight threshold is set to 0.019. Different colors of the nodes indicate different countries. The red edges indicate cross-country relationships while the gray edges indicate domestic relationships. The edge width is proportional to the edge weight, i.e., the share of the value-added contribution. The codes of countries and industries can be found in S1 and S2 Tables.
(PDF)

S4 Fig The GVT rooted at South Korea’s transport equipment industry in 2003. The edge weight threshold is set to 0.019. Different colors of the nodes indicate different countries. The red edges indicate cross-country relationships while the gray edges indicate domestic relationships. The edge width is proportional to the edge weight, i.e., the share of the value-added contribution. The codes of countries and industries can be found in S1 and S2 Tables.
(PDF)

S5 Fig The GVT rooted at South Korea’s transport equipment industry in 2011. The edge weight threshold is set to 0.019. Different colors of the nodes indicate different countries. The red edges indicate cross-country relationships while the gray edges indicate domestic relationships. The edge width is proportional to the edge weight, i.e., the share of the value-added contribution. The codes of countries and industries can be found in S1 and S2 Tables.
(PDF)

S6 Fig The GVT rooted at Japan’s transport equipment industry in 1995. The edge weight threshold is set to 0.019. Different colors of the nodes indicate different countries. The red edges indicate cross-country relationships while the gray edges indicate domestic relationships. The edge width is proportional to the edge weight, i.e., the share of the value-added contribution. The codes of countries and industries can be found in S1 and S2 Tables.
(PDF)

S7 Fig The GVT rooted at Indonesia’s transport equipment industry in 1995. The edge weight threshold is set to 0.019. Different colors of the nodes indicate different countries. The red edges indicate cross-country relationships while the gray edges indicate domestic relationships. The edge width is proportional to the edge weight, i.e., the share of the value-added contribution. The codes of countries and industries can be found in S1 and S2 Tables.
(PDF)

S1 Table List of WIOD economies. (PDF)

S2 Table List of WIOD industries. (PDF)

S3 Table The top-20 industries identified by the tree-based importance measure and other network centrality measures for the selected years. *TI* is the tree-based importance measure, *CC* is the closeness centrality, *BC* is the betweenness centrality, *PR* is the PageRank centrality, *VT* is the industry total value-added. The codes of countries and industries can be found in S1 and S2 Tables.
(PDF)

S4 Table The country rankings based on the tree-based importance measure and other network centrality measures for the selected years. *TI* is the tree-based importance measure, *CC* is the closeness centrality, *BC* is the betweenness centrality, *PR* is the PageRank centrality, *VT* is the industry total value-added. The codes of countries can be found in S1 Table.
(PDF)
