# Supplementary figures and images for: Global Value Trees
Source: PLoS One. 2015 May 15;10(5):e0126699. doi: 10.1371/journal.pone.0126699 (PMC4433196; doi:10.1371/journal.pone.0126699)

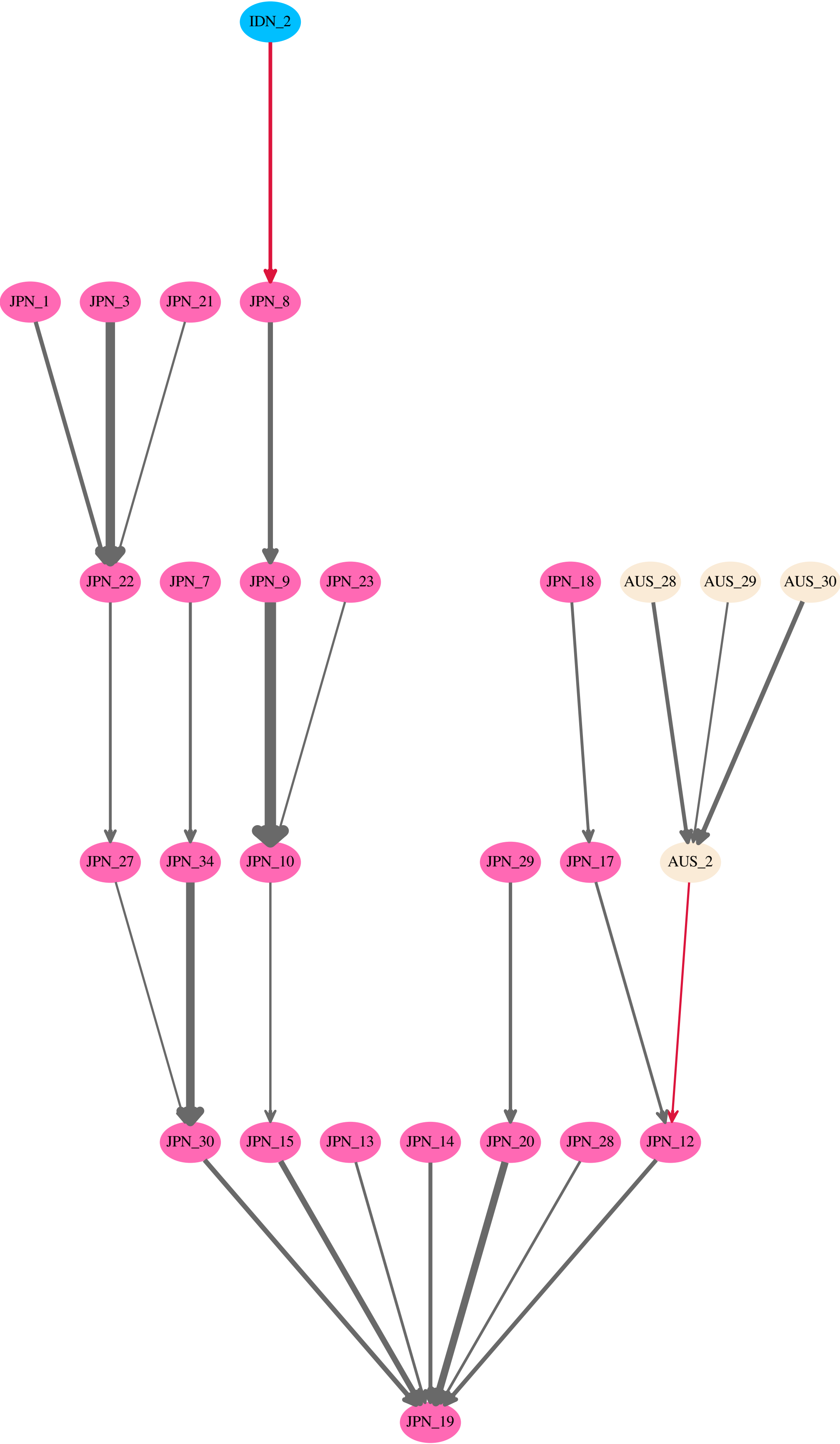

Supplement: S1 Fig — The edge weight threshold is set to 0.019. Different colors of the nodes indicate different countries. The red edges indicate cross-country relationships while the gray edges indicate domestic relationships. The edge width is proportional to the edge weight, i.e., the share of the value-added contribution. The codes of countries and industries can be found in S1 and S2 Tables. (PDF) [file pone.0126699.s001.pdf]

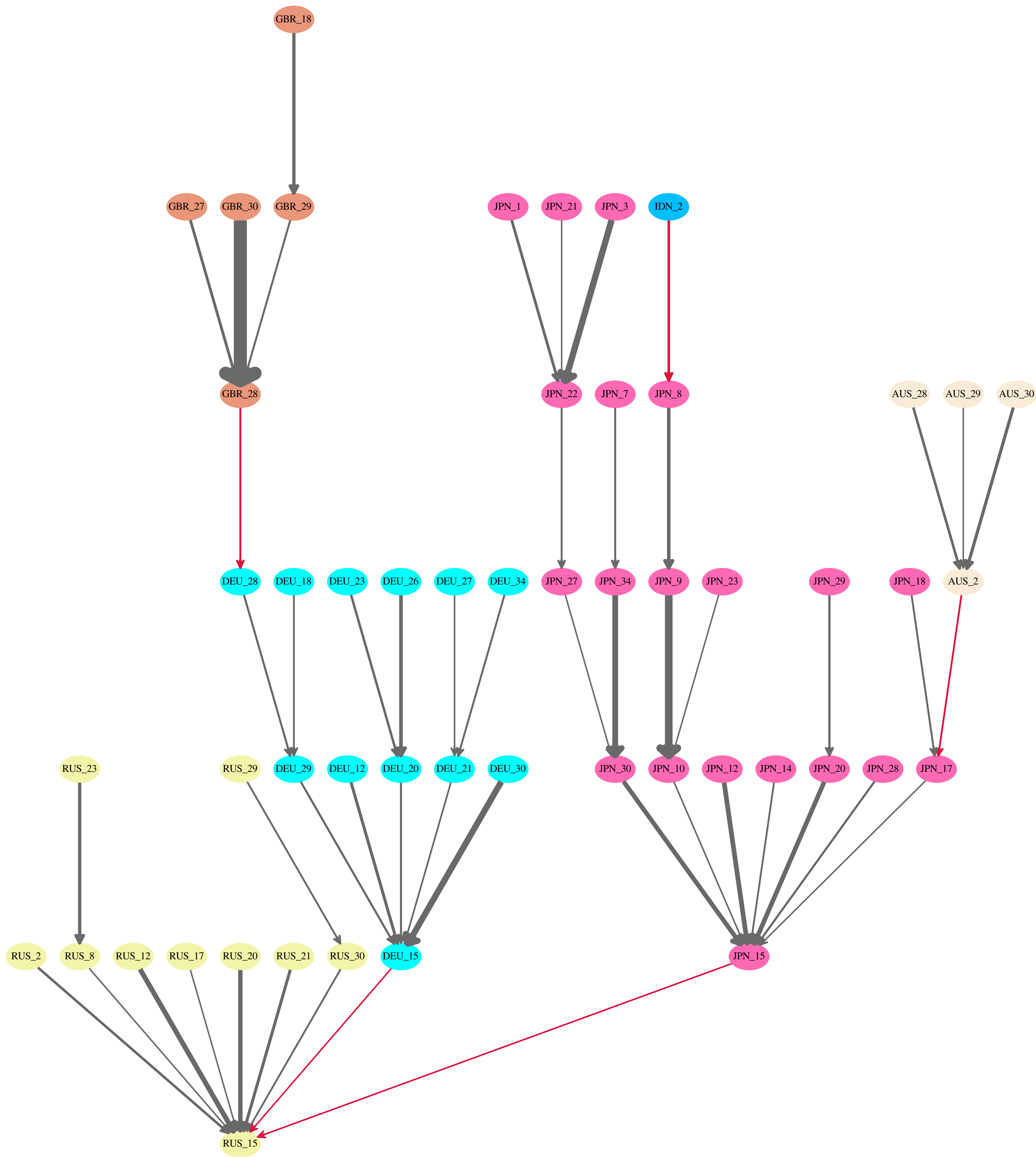

Supplement: S2 Fig — The edge weight threshold is set to 0.019. Different colors of the nodes indicate different countries. The red edges indicate cross-country relationships while the gray edges indicate domestic relationships. The edge width is proportional to the edge weight, i.e., the share of the value-added contribution. The codes of countries and industries can be found in S1 and S2 Tables. (PDF) [file pone.0126699.s002.pdf]

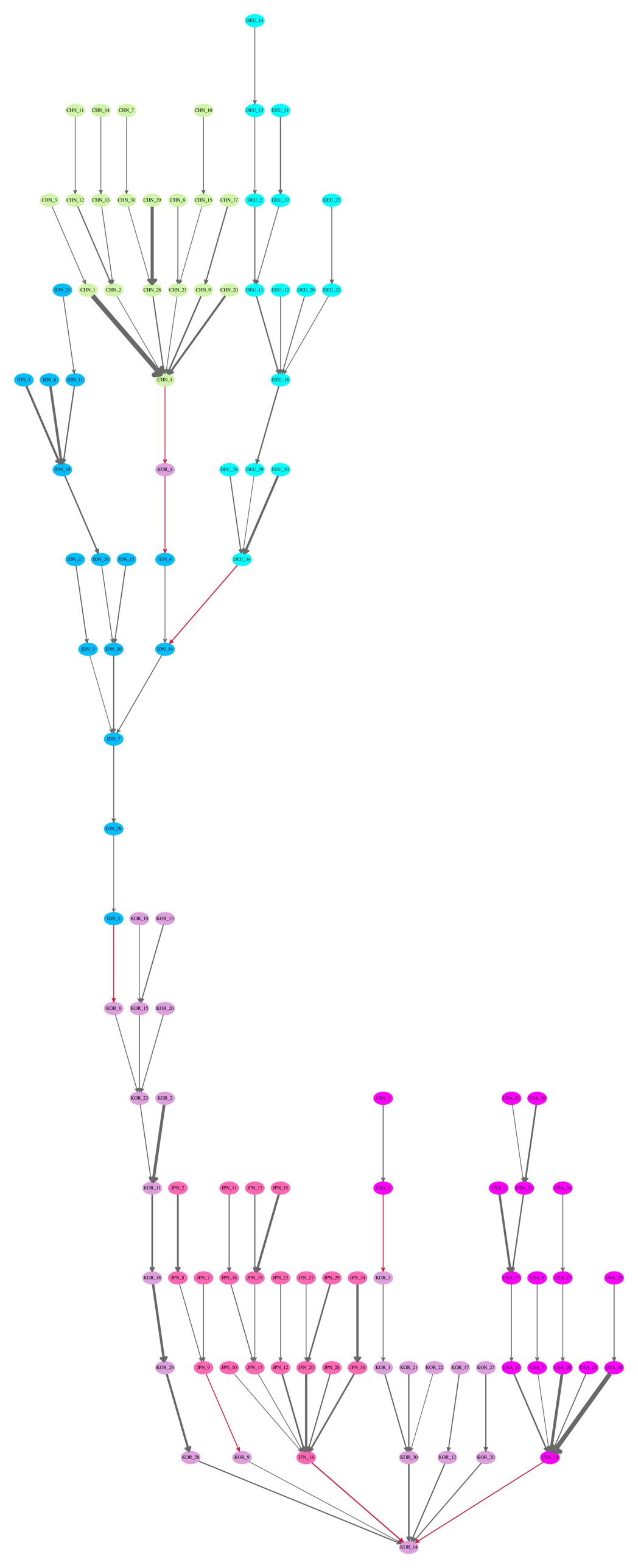

Supplement: S3 Fig — The edge weight threshold is set to 0.019. Different colors of the nodes indicate different countries. The red edges indicate cross-country relationships while the gray edges indicate domestic relationships. The edge width is proportional to the edge weight, i.e., the share of the value-added contribution. The codes of countries and industries can be found in S1 and S2 Tables. (PDF) [file pone.0126699.s003.pdf]

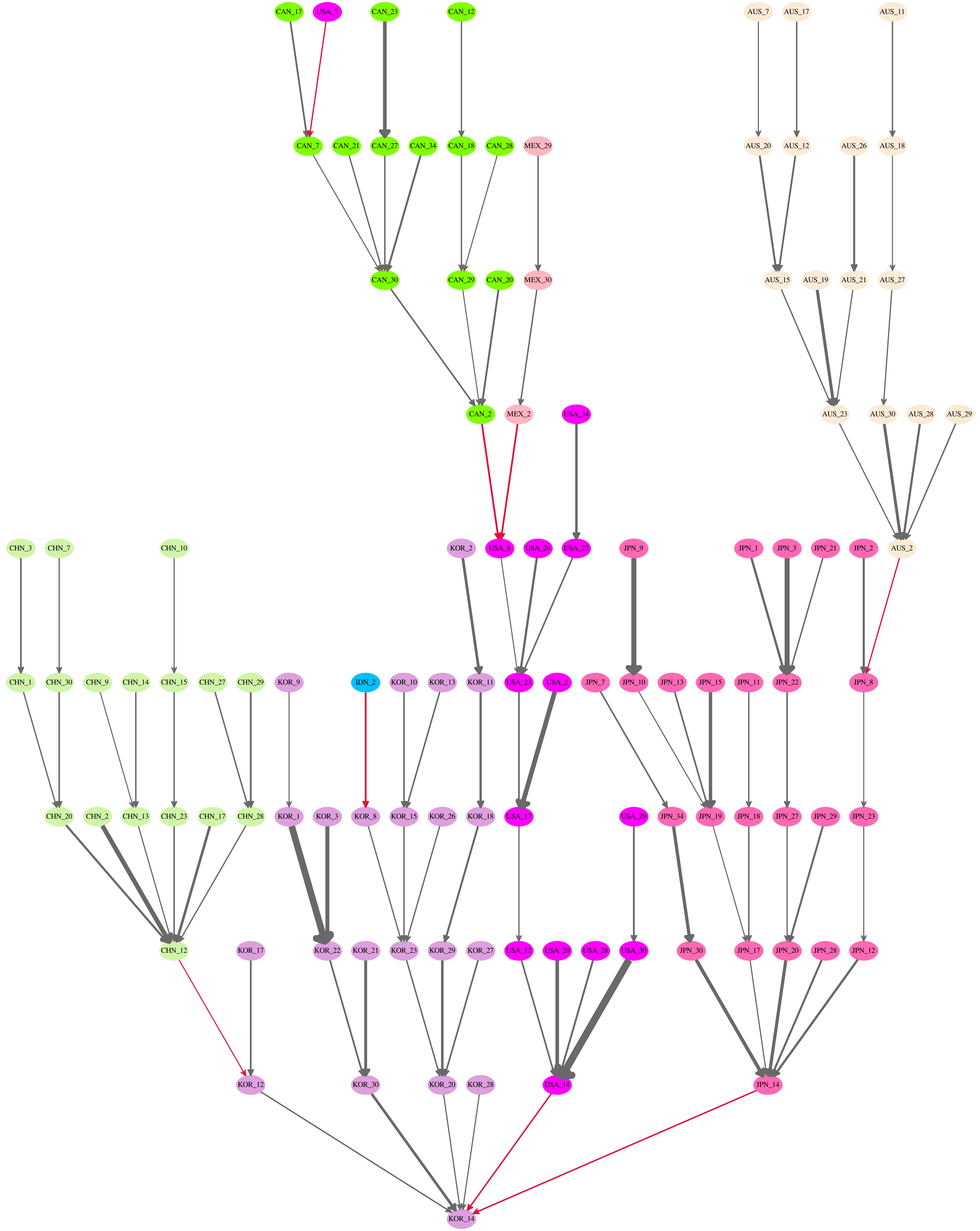

Supplement: S4 Fig — The edge weight threshold is set to 0.019. Different colors of the nodes indicate different countries. The red edges indicate cross-country relationships while the gray edges indicate domestic relationships. The edge width is proportional to the edge weight, i.e., the share of the value-added contribution. The codes of countries and industries can be found in S1 and S2 Tables. (PDF) [file pone.0126699.s004.pdf]

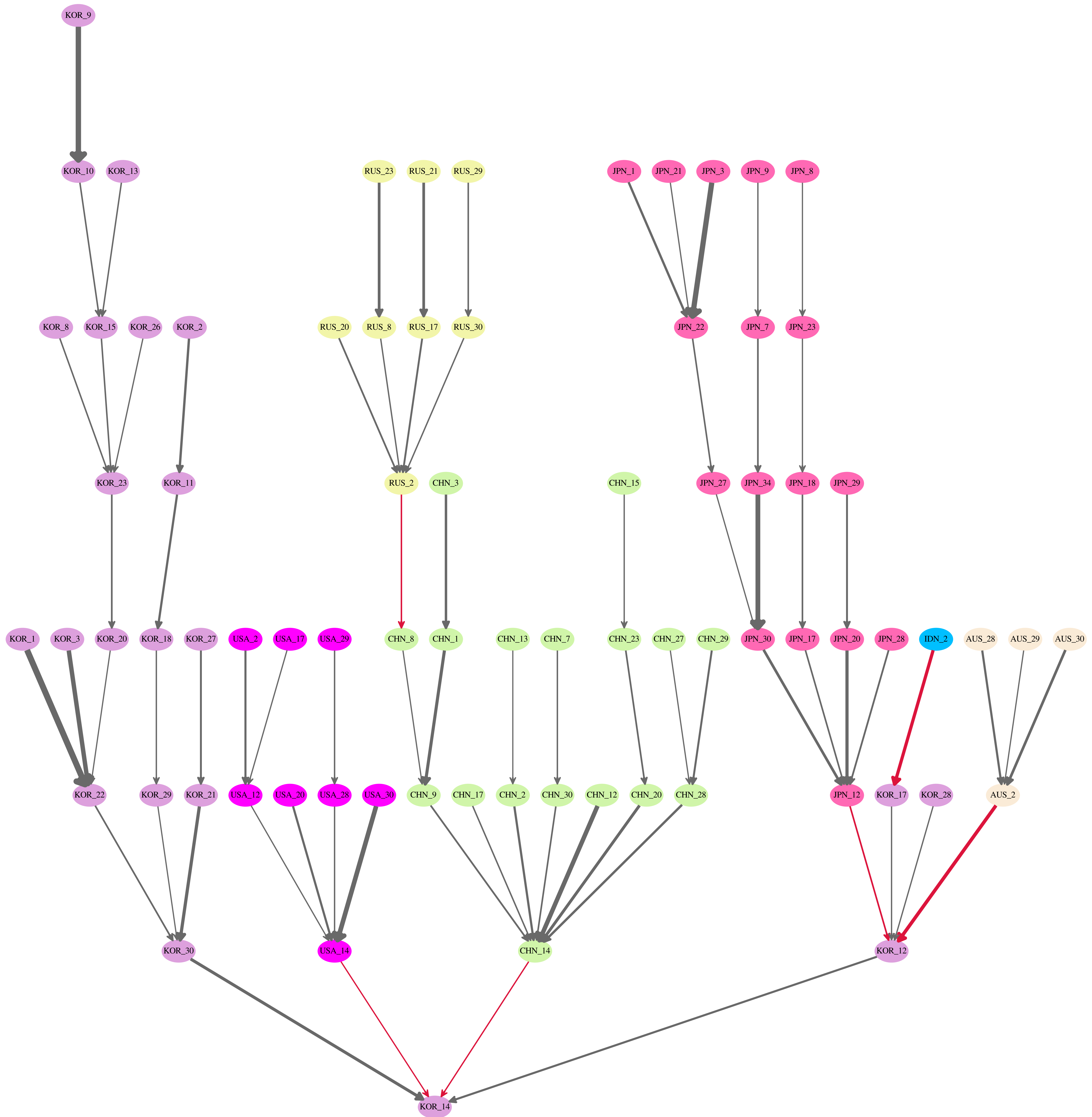

Supplement: S5 Fig — The edge weight threshold is set to 0.019. Different colors of the nodes indicate different countries. The red edges indicate cross-country relationships while the gray edges indicate domestic relationships. The edge width is proportional to the edge weight, i.e., the share of the value-added contribution. The codes of countries and industries can be found in S1 and S2 Tables. (PDF) [file pone.0126699.s005.pdf]

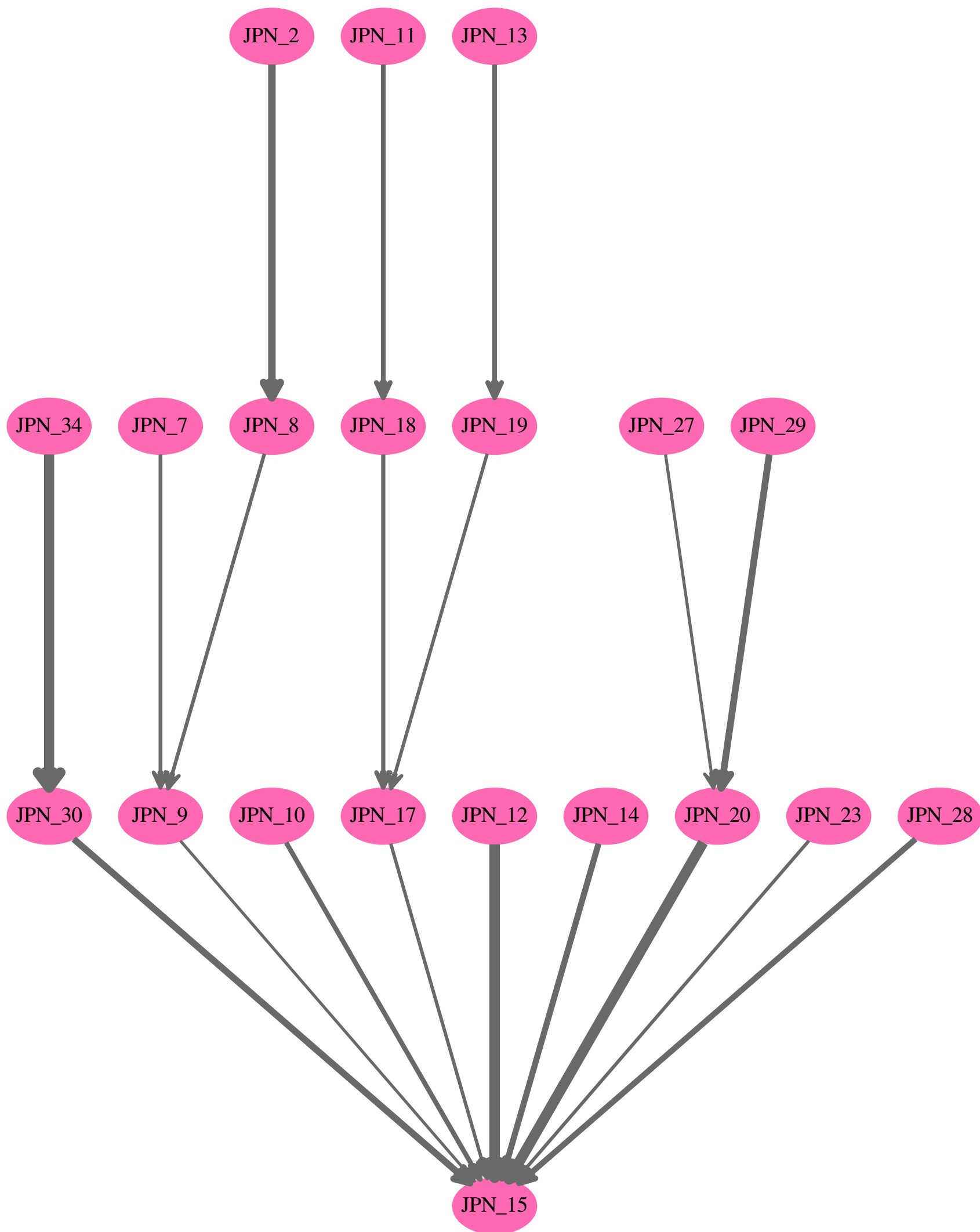

Supplement: S6 Fig — The edge weight threshold is set to 0.019. Different colors of the nodes indicate different countries. The red edges indicate cross-country relationships while the gray edges indicate domestic relationships. The edge width is proportional to the edge weight, i.e., the share of the value-added contribution. The codes of countries and industries can be found in S1 and S2 Tables. (PDF) [file pone.0126699.s006.pdf]

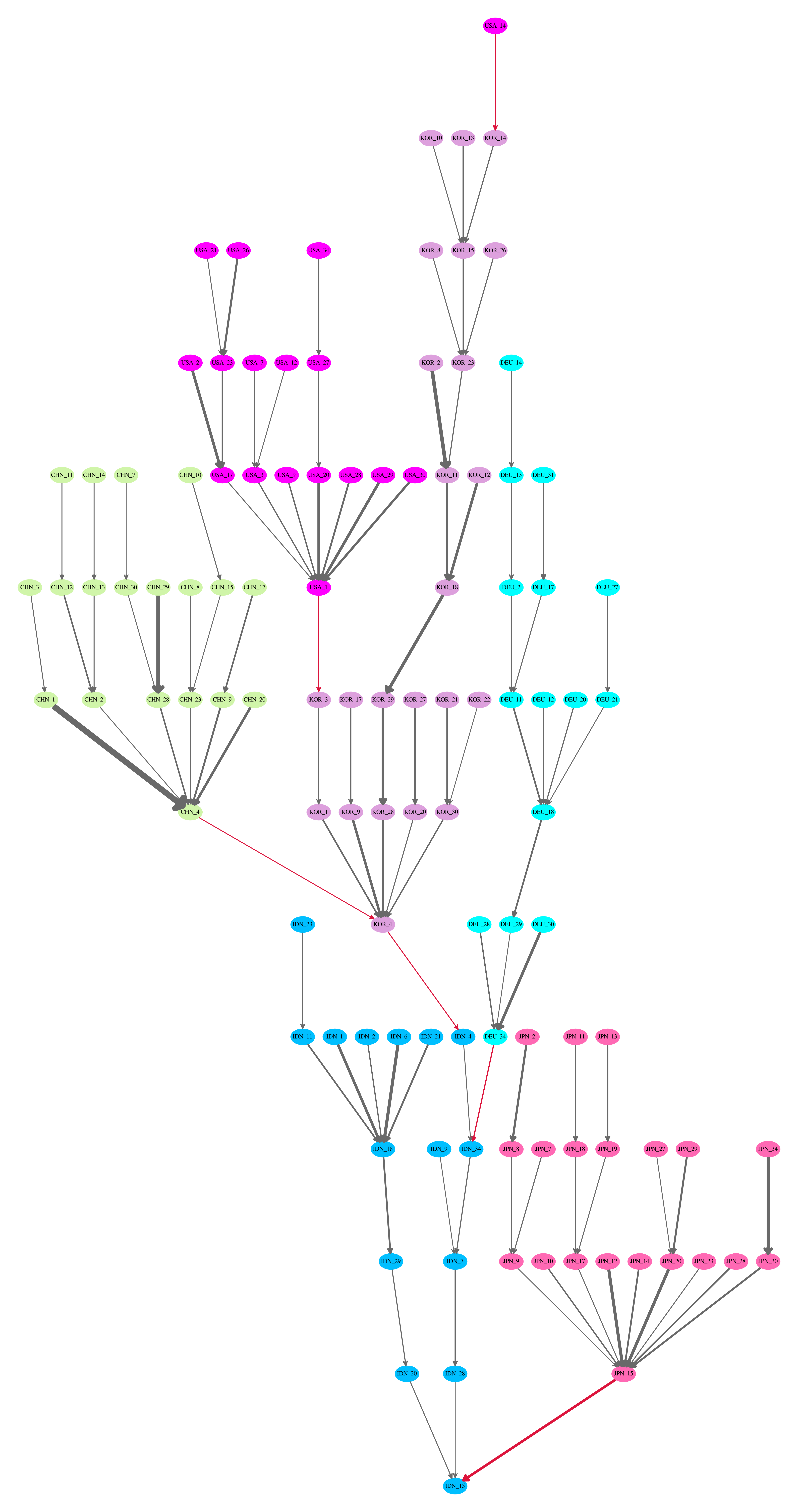

Supplement: S7 Fig — The edge weight threshold is set to 0.019. Different colors of the nodes indicate different countries. The red edges indicate cross-country relationships while the gray edges indicate domestic relationships. The edge width is proportional to the edge weight, i.e., the share of the value-added contribution. The codes of countries and industries can be found in S1 and S2 Tables. (PDF) [file pone.0126699.s007.pdf]
