# Supplementary material for: Global Value Trees
Source: PLoS One. 2015 May 15;10(5):e0126699. doi: 10.1371/journal.pone.0126699 (PMC4433196; doi:10.1371/journal.pone.0126699)
Supplement: S1 Table — (PDF) [file pone.0126699.s008.pdf]

Euro-Zone		Non-Euro EU		NAFTA		East Asia		BRIIAT	
**Economy**	**3L Code**	**Economy**	**3L Code**	**Economy**	**3L Code**	**Economy**	**3L Code**	**Economy**	**3L Code**
Austria	AUT	Bulgaria	BGR	Canada	CAN	China	CHN	Australia	AUS
Belgium	BEL	Czech Rep.	CZE	Mexico	MEX	Japan	JPN	Brazil	BRA
Cyprus	CYP	Denmark	DNK	USA	USA	South Korea	KOR	India	IND
Estonia	EST	Hungary	HUN			Taiwan	TWN	Indonesia	IDN
Finland	FIN	Latvia	LVA					Russia	RUS
France	FRA	Lithuania	LTU					Turkey	TUR
Germany	DEU	Poland	POL						
Greece	GRC	Romania	ROM						
Ireland	IRL	Sweden	SWE						
Italy	ITA	UK	GBR						
Luxembourg	LUX								
Malta	MLT								
Netherlands	NLD								
Portugal	PRT								
Slovakia	SVK								
Slovenia	SVN								
Spain	ESP								
