# Supplementary material for: Global Value Trees
Source: PLoS One. 2015 May 15;10(5):e0126699. doi: 10.1371/journal.pone.0126699 (PMC4433196; doi:10.1371/journal.pone.0126699)
Supplement: S2 Table — (PDF) [file pone.0126699.s009.pdf]

**Full Name**	**ISIC Rev. 3 Code**	**WIOD Code**	**3-Letter Code**
Agriculture, Hunting, Forestry and Fishing	AtB	c1	Agr
Mining and Quarrying	C	c2	Min
Food, Beverages and Tobacco	15t16	c3	Fod
Textiles and Textile Products	17t18	c4	Tex
Leather, Leather and Footwear	19	c5	Lth
Wood and Products of Wood and Cork	20	c6	Wod
Pulp, Paper, Paper , Printing and Publishing	21t22	c7	Pup
Coke, Refined Petroleum and Nuclear Fuel	23	c8	Cok
Chemicals and Chemical Products	24	c9	Chm
Rubber and Plastics	25	c10	Rub
Other Non-Metallic Mineral	26	c11	Omn
Basic Metals and Fabricated Metal	27t28	c12	Met
Machinery, Nec	29	c13	Mch
Electrical and Optical Equipment	30t33	c14	Elc
Transport Equipment	34t35	c15	Tpt
Manufacturing, Nec; Recycling	36t37	c16	Mnf
Electricity, Gas and Water Supply	E	c17	Ele
Construction	F	c18	Cst
Sale, Maintenance and Repair of Motor Vehicles and Motorcycles; Retail Sale of Fuel	50	c19	Sal
Wholesale Trade and Commission Trade, Except of Motor Vehicles and Motorcycles	51	c20	Whl
Retail Trade, Except of Motor Vehicles and Motorcycles; Repair of Household Goods	52	c21	Rtl
Hotels and Restaurants	H	c22	Htl
Inland Transport	60	c23	Ldt
Water Transport	61	c24	Wtt
Air Transport	62	c25	Ait
Other Supporting and Auxiliary Transport Activities; Activities of Travel Agencies	63	c26	Otr
Post and Telecommunications	64	c27	Pst
Financial Intermediation	J	c28	Fin
Real Estate Activities	70	c29	Est
Renting of M&Eq and Other Business Activities	71t74	c30	Obs
Public Admin and Defence; Compulsory Social Security	L	c31	Pub
Education	M	c32	Edu
Health and Social Work	N	c33	Hth
Other Community, Social and Personal Services	O	c34	Ocm
Private Households with Employed Persons	P	c35	Pvt
