# Supplementary material for: Global Value Trees
Source: PLoS One. 2015 May 15;10(5):e0126699. doi: 10.1371/journal.pone.0126699 (PMC4433196; doi:10.1371/journal.pone.0126699)
Supplement: S3 Table — TI is the tree-based importance measure, CC is the closeness centrality, BC is the betweenness centrality, PR is the PageRank centrality, VT is the industry total value-added. The codes of countries and industries can be found in S1 and S2 Tables. (PDF) [file pone.0126699.s010.pdf]

Rank	1995					2003					2011				
	TI	CC	BC	PR	VT	TI	CC	BC	PR	VT	TI	CC	BC	PR	VT
**1**	USA-Obs	DEU-Tpt	USA-Obs	USA-Pub	USA-Pub	USA-Ldt	USA-Obs	USA-Obs	USA-Pub	USA-Obs	USA-Fin	CHN-Cst	CHN-Cst	USA-Pub	USA-Obs
**2**	USA-Pst	DEU-Obs	DEU-Obs	USA-Cst	USA-Est	JPN-Cok	USA-Tpt	DEU-Tpt	USA-Hth	USA-Pub	USA-Obs	CHN-Met	USA-Pub	CHN-Cst	USA-Pub
**3**	JPN-Ele	DEU-Est	USA-Pub	USA-Hth	USA-Obs	USA-Cok	USA-Pub	USA-Tpt	USA-Cst	USA-Est	USA-Est	CHN-Omn	USA-Obs	USA-Hth	USA-Est
**4**	JPN-Obs	DEU-Cst	USA-Elc	JPN-Cst	USA-Rtl	AUS-Min	USA-Hth	DEU-Obs	USA-Est	USA-Fin	RUS-Min	CHN-Min	RUS-Min	CHN-Pub	USA-Fin
**5**	USA-Whl	DEU-Elc	DEU-Tpt	USA-Est	USA-Fin	USA-Ele	USA-Fin	USA-Pub	USA-Tpt	USA-Rtl	CHN-Cok	CHN-Whl	DEU-Obs	DEU-Tpt	USA-Hth
**6**	JPN-Sal	DEU-Fod	RUS-Min	USA-Tpt	JPN-Est	USA-Obs	USA-Cst	DEU-Cok	CHN-Cst	USA-Hth	AUS-Min	CHN-Elc	DEU-Tpt	CHN-Elc	USA-Rtl
**7**	JPN-Ocm	USA-Obs	FRA-Obs	USA-Htl	USA-Whl	USA-Met	USA-Est	JPN-Tpt	DEU-Tpt	USA-Whl	DEU-Est	CHN-Fin	CHN-Elc	USA-Cst	USA-Whl
**8**	DEU-Cst	USA-Pub	JPN-Elc	USA-Rtl	USA-Hth	USA-Fin	USA-Rtl	DEU-Elc	USA-Htl	USA-Cst	JPN-Obs	CHN-Ldt	CHN-Agr	CHN-Hth	JPN-Est
**9**	DEU-Est	FRA-Obs	USA-Fod	DEU-Cst	JPN-Whl	JPN-Ldt	USA-Whl	USA-Elc	USA-Rtl	JPN-Est	CHN-Ldt	CHN-Agr	GBR-Obs	USA-Htl	CHN-Agr
**10**	USA-Ocm	DEU-Hth	DEU-Met	USA-Fod	JPN-Cst	CAN-Min	USA-Ocm	USA-Fod	USA-Ocm	USA-Ocm	GBR-Fin	CHN-Ele	ESP-Obs	USA-Tpt	USA-Ocm
**11**	JPN-Ldt	DEU-Met	USA-Tpt	DEU-Tpt	USA-Cst	USA-Est	USA-Htl	JPN-Whl	USA-Fod	USA-Htl	DEU-Fin	CHN-Chm	FIN-Obs	USA-Rtl	USA-Cst
**12**	USA-Fin	USA-Elc	JPN-Whl	DEU-Fod	JPN-Fin	JPN-Ele	USA-Fod	RUS-Min	GBR-Pub	JPN-Pub	USA-Cok	CHN-Fod	USA-Fin	USA-Est	CHN-Whl
**13**	USA-Est	USA-Whl	JPN-Cst	USA-Ocm	JPN-Pub	DEU-Est	USA-Elc	JPN-Cst	JPN-Cst	JPN-Whl	CHN-Chm	CHN-Obs	USA-Cok	CHN-Mch	JPN-Pub
**14**	DEU-Omn	USA-Tpt	DEU-Elc	JPN-Pub	JPN-Obs	GBR-Fin	USA-Pst	CHN-Elc	DEU-Fod	JPN-Obs	CHN-Obs	CHN-Pst	CHN-Tex	CHN-Tpt	CHN-Cst
**15**	USA-Ele	USA-Hth	DEU-Tex	DEU-Hth	JPN-Rtl	JPN-Elc	DEU-Tpt	DEU-Met	DEU-Cst	USA-Pst	JPN-Pst	CHN-Mch	DEU-Met	USA-Fod	USA-Htl
**16**	JPN-Fin	USA-Cst	DEU-Cok	GBR-Pub	USA-Ocm	AUS-Ldt	USA-Met	ESP-Tpt	CHN-Pub	DEU-Obs	CAN-Min	CHN-Otr	FIN-Cst	USA-Ocm	JPN-Obs
**17**	JPN-Whl	USA-Fin	FRA-Ele	DEU-Htl	DEU-Est	CAN-Obs	USA-Agr	FRA-Obs	GBR-Est	JPN-Cst	CHN-Whl	USA-Obs	TUR-Tex	GBR-Hth	DEU-Obs
**18**	USA-Ldt	USA-Rtl	DEU-Cst	DEU-Est	DEU-Obs	JPN-Cst	USA-Pup	ITA-Obs	FRA-Tpt	DEU-Est	CHN-Met	USA-Pub	FRA-Obs	CHN-Fod	JPN-Whl
**19**	JPN-Cst	USA-Est	JPN-Met	CHN-Cst	JPN-Ocm	CHN-Elc	USA-Chm	CHN-Cst	USA-Fin	JPN-Fin	JPN-Ele	USA-Hth	AUS-Min	CHN-Edu	CHN-Est
**20**	JPN-Chm	DEU-Fin	DEU-Fod	FRA-Tpt	USA-Pst	JPN-Met	DEU-Obs	USA-Agr	ESP-Cst	GBR-Obs	JPN-Htl	CHN-Wod	IDN-Min	JPN-Cst	CHN-Met
