# Supplementary material for: Global Value Trees
Source: PLoS One. 2015 May 15;10(5):e0126699. doi: 10.1371/journal.pone.0126699 (PMC4433196; doi:10.1371/journal.pone.0126699)
Supplement: S4 Table — TI is the tree-based importance measure, CC is the closeness centrality, BC is the betweenness centrality, PR is the PageRank centrality, VT is the industry total value-added. The codes of countries can be found in S1 Table. (PDF) [file pone.0126699.s011.pdf]

	1995					2003					2011				
**Rank**	*TI*	*CC*	*BC*	*PR*	*VT*	*TI*	*CC*	*BC*	*PR*	*VT*	*TI*	*CC*	*BC*	*PR*	*VT*
**1**	**JPN**	**USA**	**DEU**	**USA**	**USA**	**USA**	**USA**	**USA**	**USA**	**USA**	**USA**	**USA**	**CHN**	**USA**	**USA**
**2**	**USA**	**FRA**	**USA**	**DEU**	**JPN**	**JPN**	**FRA**	**DEU**	**DEU**	**JPN**	**CHN**	**FRA**	**USA**	**CHN**	**CHN**
**3**	**DEU**	**CAN**	**JPN**	**JPN**	**DEU**	**AUS**	**MEX**	**JPN**	**GBR**	**DEU**	**JPN**	**CAN**	**DEU**	**DEU**	**JPN**
**4**	**IDN**	**MEX**	**FRA**	**GBR**	**FRA**	**CAN**	**CAN**	**CHN**	**CHN**	**GBR**	**RUS**	**MEX**	**RUS**	**GBR**	**DEU**
**5**	**KOR**	**GBR**	**RUS**	**ITA**	**GBR**	**DEU**	**GBR**	**ITA**	**ITA**	**FRA**	**DEU**	**GBR**	**ESP**	**ITA**	**FRA**
**6**	**GBR**	**TWN**	**ITA**	**FRA**	**ITA**	**GBR**	**DEU**	**ESP**	**JPN**	**CHN**	**CAN**	**JPN**	**GBR**	**FRA**	**GBR**
**7**	**FRA**	**DEU**	**GBR**	**ESP**	**CHN**	**CHN**	**JPN**	**RUS**	**FRA**	**ITA**	**AUS**	**DEU**	**FIN**	**JPN**	**BRA**
**8**	**CHN**	**JPN**	**ESP**	**KOR**	**BRA**	**ITA**	**ITA**	**FRA**	**ESP**	**ESP**	**GBR**	**BRA**	**KOR**	**RUS**	**ITA**
**9**	**ITA**	**ITA**	**FIN**	**CHN**	**ESP**	**ESP**	**ESP**	**FIN**	**KOR**	**CAN**	**IND**	**ITA**	**AUS**	**ESP**	**IND**
**10**	**ESP**	**KOR**	**AUS**	**GRC**	**CAN**	**FRA**	**KOR**	**GBR**	**TUR**	**MEX**	**ESP**	**RUS**	**BRA**	**BRA**	**CAN**
11	NLD	ESP	BRA	BRA	KOR	KOR	IND	IND	CAN	KOR	ITA	IND	JPN	CAN	ESP
12	BRA	AUS	ROM	RUS	NLD	IND	AUS	TUR	MEX	IND	FRA	AUS	FRA	IND	AUS
13	CAN	BRA	DNK	AUS	IND	MEX	NLD	AUS	GRC	BRA	BRA	KOR	CAN	TUR	RUS
14	AUS	NLD	IDN	TUR	AUS	RUS	BRA	IDN	AUS	AUS	KOR	ESP	ITA	KOR	MEX
15	RUS	RUS	KOR	NLD	RUS	BRA	TWN	BRA	RUS	NLD	IDN	TUR	IDN	AUS	KOR
16	TWN	IND	BEL	IND	MEX	IDN	RUS	DNK	IND	RUS	BEL	TWN	TUR	GRC	IDN
17	MEX	BEL	IND	CAN	BEL	SWE	BEL	POL	NLD	TWN	MEX	NLD	MEX	MEX	NLD
18	BEL	DNK	TUR	SWE	TWN	AUT	TUR	HUN	SWE	BEL	TUR	BEL	GRC	POL	TUR
19	AUT	SWE	CHN	AUT	IDN	NLD	SWE	BGR	BRA	SWE	NLD	POL	LVA	IDN	SWE
20	SWE	POL	MEX	BEL	SWE	BEL	POL	ROM	POL	TUR	AUT	FIN	CZE	NLD	BEL
21	DNK	FIN	BGR	DNK	AUT	TUR	GRC	CAN	AUT	IDN	SWE	GRC	HUN	SWE	POL
22	TUR	AUT	GRC	PRT	TUR	PRT	DNK	KOR	BEL	AUT	POL	PRT	POL	BEL	TWN
23	IND	PRT	NLD	TWN	DNK	DNK	PRT	SVK	IDN	POL	GRC	AUT	IND	FIN	AUT
24	PRT	GRC	CAN	MEX	POL	TWN	AUT	GRC	FIN	DNK	PRT	DNK	SVK	AUT	DNK
25	GRC	CZE	LVA	FIN	GRC	POL	FIN	TWN	DNK	GRC	TWN	IRL	CYP	ROM	GRC
26	POL	IRL	HUN	IDN	FIN	GRC	IRL	PRT	PRT	FIN	DNK	ROM	SWE	DNK	FIN
27	FIN	CHN	PRT	POL	PRT	FIN	CHN	EST	IRL	IRL	FIN	CZE	ROM	PRT	PRT
28	IRL	IDN	POL	CZE	IRL	CZE	CZE	BEL	CZE	PRT	CZE	HUN	PRT	CZE	IRL
29	CZE	ROM	SWE	HUN	CZE	IRL	IDN	CZE	HUN	CZE	ROM	SVK	DNK	CYP	CZE
30	HUN	TUR	TWN	ROM	HUN	ROM	HUN	LVA	CYP	HUN	IRL	BGR	SVN	HUN	ROM
31	ROM	HUN	AUT	CYP	ROM	HUN	ROM	SWE	TWN	ROM	SVK	CHN	BEL	TWN	HUN
32	SVN	SVN	CYP	IRL	LUX	SVK	SVK	CYP	ROM	SVK	HUN	IDN	BGR	IRL	SVK
33	SVK	BGR	CZE	SVN	SVN	SVN	SVN	MEX	SVN	LUX	BGR	SWE	MLT	BGR	LUX
34	CYP	SVK	LTU	BGR	SVK	BGR	LTU	LTU	LTU	SVN	SVN	LTU	LUX	SVK	BGR
35	BGR	LUX	SVN	LVA	BGR	LTU	BGR	MLT	LVA	BGR	CYP	SVN	LTU	LVA	SVN
36	LTU	LTU	IRL	LTU	CYP	CYP	LUX	NLD	SVK	LTU	LVA	EST	EST	LTU	LTU
37	LVA	CYP	EST	SVK	LTU	EST	CYP	SVN	BGR	CYP	LTU	LVA	TWN	SVN	LVA
38	EST	EST	SVK	EST	LVA	LVA	LVA	AUT	EST	LVA	EST	LUX	NLD	EST	CYP
39	LUX	MLT	MLT	MLT	EST	MLT	EST	IRL	MLT	EST	LUX	CYP	AUT	MLT	EST
